# Who benefits from multifamily psychoeducation groups ? Descriptive anaysis of participants

**DOI:** 10.1192/j.eurpsy.2024.322

**Published:** 2024-08-27

**Authors:** O. Amiot, C. Genis, L. Baziret, L. Champlon, S. Saïd

**Affiliations:** 92100, GH Paul Guiraud, Boulogne Billancourt, France

## Abstract

**Introduction:**

Guidelines for relapse prevention in schizophrenia recommend psychoeducation for patients and caregivers (Bighelli I, Leucht S et al. Lancet Psychiatry 2021). Considering that, in 2021, we implemented in our Psychiatric community center a multifamily therapy (MFT). The program is based on systemic approach and psychoeducation, focusing on schizophrenia.

**Objectives:**

Describe participants of MFT groups focusing on schizophrenia.

* Patients’ characteristics : age, gender, duration of psychiatric follow-up, history of hospitalization

* Caregivers’ characterics: status, age.

**Methods:**

We carried out a descriptive study of the different profile of MFT groups participants in our community center from 2021 to today.

**Results:**

Since 2021, 4 MFT groups took place including 50 participants: 18 patients suffering from schizophrenia and 32 relatives.

Image 1 illustrates the different participants of each group.

Each group was different. Some patients came with both their parents, even if divorced, some came only with their mother. Some came with a sibling. Nevertheless, the numbers of fathers and siblings did not always allow us to work in sub-groups.

Considering patients: 18 patients benefited from our program. 8 female and 10 male patients (55.6%) were admitted and distributed in each group as described in image 2. The mean age of patients was 31.9 years old [20.1 – 57.5]. Each group was made up of patients with psychiatric follow-up ranging from 1 year to more than 20 years, and having experienced between 1 to more than 5 psychiatric hospitalizations. It appears that Group 4 was noticeably younger than the other groups with a mean age of 22.4 years old [20.4 – 26.7] and a shorter history in psychiatry with less hospitalisations (image 3).

Considering relatives: 15 mothers, 9 fathers, 5 siblings, 1 spouse, 1 aunt and 1 uncle benefited from psychoeducation to caregivers. The relatives were from 47 to 81 years old for the parents, and from 17 to 50,7 for the siblings. Unlike parents, siblings generally attended a limited number of sessions.

**Image:**

**Figure:**
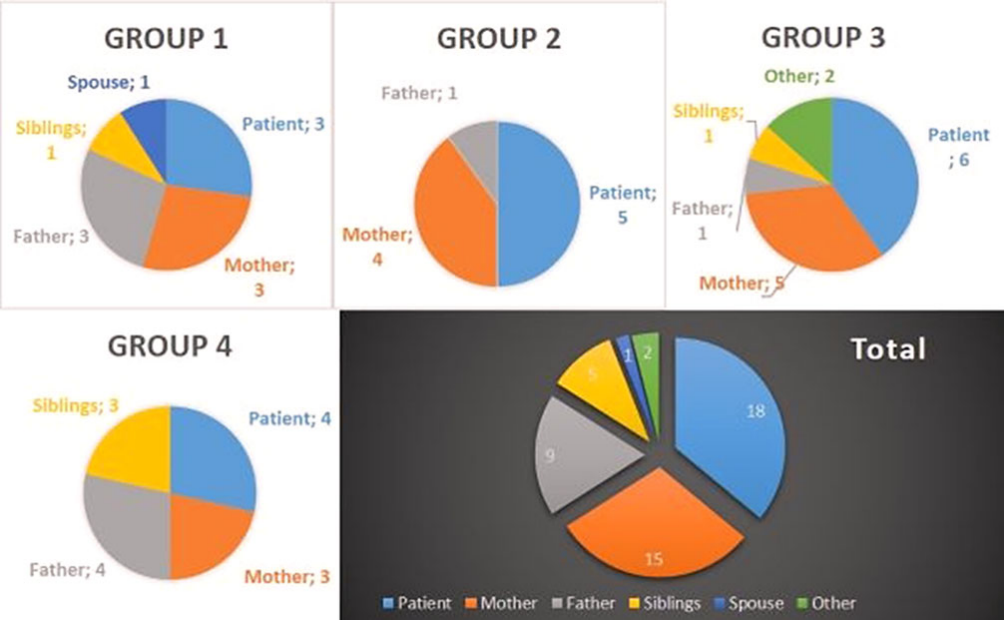

**Image 2:**

**Figure:**
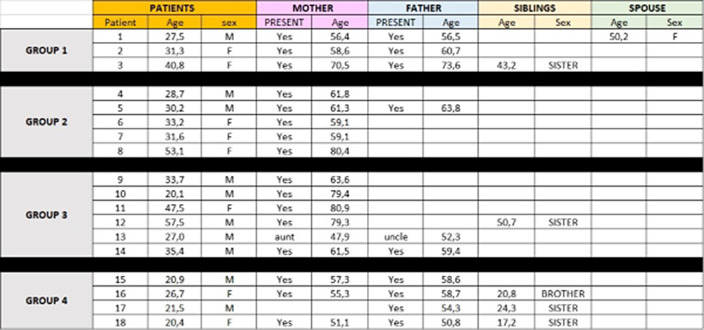

**Image 3:**

**Figure:**
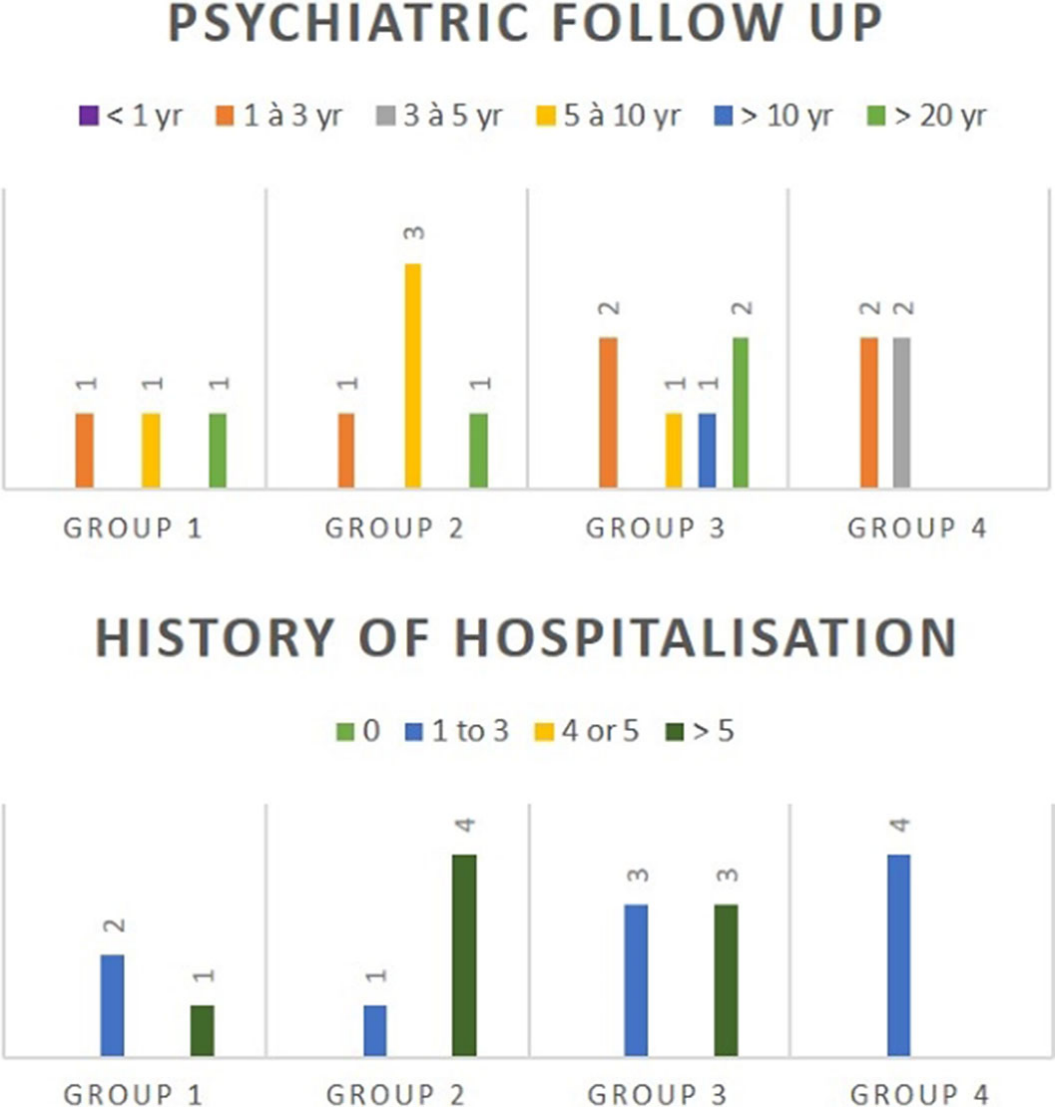

**Conclusions:**

This descriptive study reflects the work carried out with 18 patients and their relatives in an MFT group providing psychoeducation to patients suffering from schizophrenia and their caregivers. 50 persons benefited from psychoeducation in 2 years. We learned from these results to improve the constitution of our groups and the benefits of our psychoeducation program. We were careful to include families with siblings as we know they are affected by the mental illness in the family and are often left aside of all care/support proposals. We questioned ourselves on the advantages of homogeneous or heterogeneous groups, considering age, history of follow up. How could it impact affiliation to the group or differentiation movements ? How useful or harmful it is for sharing experiences between the families. A proper study would be necessary to answer these questions.

**Disclosure of Interest:**

None Declared

